# Supplementary material for: Marker-Assisted Improvement for Durable Bacterial Blight Resistance in Aromatic Rice Cultivar HUR 917 Popular in Eastern Parts of India
Source: Plants (Basel). 2023 Mar 18;12(6):1363. doi: 10.3390/plants12061363 (PMC10058408; doi:10.3390/plants12061363)
Supplement: Supplementary file 1 [file plants-12-01363-s001.zip › plants-2075944-supplementary.pdf]

Title page

'Marker-assisted improvement for durable bacterial blight resistance in aromatic rice cultivar HUR 917 popular in eastern parts of India'

Manish Kumar[1], Ravi Pratap Singh[1], Debarchana Jena[2], Vineeta Singh[2], Diptibala Rout[2], Panduranga B. Arsode[1], Madhu Choudhary[1], Prakash Singh[3], Suman Chahar[5], Sanghamitra Samantaray[2], Arup Kumar Mukherjee[2], Chander Mohan[6], Abhishek Bohra[7], Goutam Das[2], Sumana Balo[8], Onkar Nath Singh[4], Ramlakhan Verma[2*]

[1] Institute of Agricultural Science, Banaras Hindu University, Varanasi 221005, Uttar Pradesh
[2] ICAR-National Rice Research Institute, Cuttack 753006, Odisha, India
[3] Veer Kunwar Singh College of Agriculture, Bihar Agricultural University-BAU, Sabaur, Dumraon, Buxar 802136, Bihar, India
[4] Central State Farm, National Seeds Corporation, Sirsa Road, Hisar 125001, Haryana, India
[5] Department of Agriculture, Cooperation and Farmers Welfare, Government of India, New Delhi 110001, India
[6] State Agricultural Biotechnology Centre (SABC) and Centre for Crop and Food Innovation (CCFI), Murdoch University, Perth, WA *6150,* Australia
[7] Department of Soil Science and Agricultural Chemistry, Uttar Banga Krishi Vishwavidyalaya, Coochbehar 736165, West Bengal, India
[8] Birsa Agricultural University (BAU), Ranchi 834006, Jharkhand, India
* Correspondence: prakash201288@gmail.com (P.S.); ramlakhan.verma@icar.gov.in (R.V.); Tel: +91-8895237769 (P.S.); 91-8847841041 (R.V.)

Supplementary Table S1. Similarity matrix of parental lines taken for genetic diversity analysis

Genotypes	HUR 917	IRBB 66	IR64 MAS	CRMS 31B MAS	PR 114 IMP	Lalat MAS
IRBB 66	0.77					
IR64 MAS	0.76	0.06				
CRMS 31B MAS	0.50	0.71	0.65			
PR 114 improved	0.17	0.33	0.33	0.50		
Lalat MAS	0.17	0.50	0.50	0.67	0.67	

Supplementary Table S2. A list of polymorphic SSR markers between parents HUR 917 and IRBB66

Chromosome	Number of total SSRs	Number of polymorphic SSRs	Percent polymorphism	Name of the polymorphic SSR on respective chromosome
1	30	8	26.7	RM11258, RM11846, RM220, RM5443, RM11111, RM11438, RM472, RM1282,
2	30	6	20.0	RM12469, RM12983, RM13562, RM13345, RM13781, RM13996
3	30	7	23.3	RM15855, RM14320, RM14473, RM14602, RM14946, RM15441, RM15981
4	30	6	20.0	RM17263, RM17349, RM16396, RM16706, RM16820, RM6303
5	30	11	36.7	RM18530, RM19101, RM17941, RM18065, RM18270, RM18360, RM122, RM16396, RM16706, RM16820, RM3476
6	30	6	20.0	RM20724, RM20773, RM19303, RM19422, RM20096, RM420
7	30	6	20.0	RM21024, RM20913, RM20961, RM21086, RM21539, RM21879
8	30	7	23.3	RM22914, RM22321, RM22521, RM22941, RM22984, RM23356, xa13prom
9	30	5	16.7	RM23937, RM24240, RM24414, RM24448, RM215
10	30	6	20.0	RM25679, RM24999, RM25460, RM25735, RM228, RM25866
11	30	6	20.0	RM26118, RM26302, RM26393, RM26524, RM26969, pTA248
12	30	8	26.7	RM28767, RM27451, RM27840, RM28130, RM28270, RM27789, RM519, RM28766
Total	360	82	22.8%	

Supplementary Table S3. Brief summary of selection differential and bacterial blight desease index in BC and advanced segregating generations

Generation	Selection differential (Δd) for product profile trait					PDI value	AUDPC	Selection criteria
	Name of trait	HUR-917 (RP)	Range before selection	Range in selected top ten plants	Δd			
F_{1s}	-	-	-	-	-	-	-	Hybridity testing with R specific markers
BC_1F_{1s}	DFF (day)	105	88.0-114.0	100.0-108.0	1.56	11.82 ± 0.460 to 35.14 ± 0.344	204.43-599.27	FS, BS, Phenome and bioassay
	PH (cm)	111.2	93.45-111.75	102.0- 114.6	2.12			
	L/B ratio	2.962	2.16-3.812	2.752- 3.242	0.08			
	HRR (%)	69.4	53.45- 75.62	55.6-68.6	0.51			
	Aroma	2	0-2	2	0			
BC_2F_{1s}	DFF (day)	107	98.0-111.0	99.0- 108.0	1.25	5.19 ± 0.114 to 13.48 ± 0.752	93.08-191.16	FS, BS, Phenome and bioassay
	PH (cm)	108.5	90.25-108.6	104.25-113.82	2.85			
	L/B ratio	2.944	2.44-3.75	2.812 - 3.324	0.22			
	HRR (%)	63.25	54.25-77.28	59.62- 80.35	0.38			
	Aroma	2	0-2	2	0			
BC_2F_2s	DFF (day)	106	89.0-113.0	96.0-110.0	2.12	2.48 ± 0.624 to 18.24 ± 0.225	72.24-178.25	FS, BS, Phenome and bioassay analysis
	PH (cm)	113.4	85.25- 111.82	108.25-116.62	1.68			
	L/B ratio	2.895	2.082- 3.816	2.783- 3.124	0.15			
	HRR (%)	67.6	48.62-76.44	56.6 -75.8	1.53			
	Aroma	2	0-2	2	0			
BC_2F_3s	DFF (day)	103	102.0- 111.0	104.0 -114.0	0.92	2.770.87± to 8.89±1.12	79.30 - 101.63	Phenomics analysis
	PH (cm)	110.6	99.52 -110.46	108.0- 115.25	1.12			
	L/B ratio	2.91	2.65- 3.780	2.815-3.064	0.062			
	HRR (%)	65.3	48.0- 78.64	58.2-78.95	0.79			
	Aroma	2	2	2	0			
BC_2F_4s	DFF (day)	106	103.0- 114.0	104.0 – 112.0	0.62	2.68 ± 0.431 to 9.31±0.280	77.25 -105.8	BS, Phenome and bioassay analysis
	PH (cm)	110.2	100.0- 114.6	101.71-112.2	1.19			
	L/B ratio	2.965	2.752- 3.818	2.838-3.734	0.11			
	HRR (%)	68.5	52.75- 81.62	53.6-79.2	0.72			
	Aroma	2	2	2	0			

Note: FS-foreground selection, BS-background selection, PDI-percent disease index, AUDPC-area under disease progress curve, Δd-selection differential

Supplementary Table S4. RP genome recovery in BC_2F_2 generation

Near Isogenic line (NILs)	Target gene combination	Markers with RP alleles	Heterozygosity	Donor alleles	% RP genome
HR 23-5-37-83	Xa 21+ xa13+ xa5	75	4	3	93.90
HR 23-5-37-109	Xa21*+ xa13+ xa5	77	3	2	95.73
HR 23-5-37-121	Xa 21+ xa13+ xa5	75	4	3	93.90
HR 23-5-37-201	Xa 21+xa13+ xa5	77	2	3	95.12
HR 23-5-37-282	Xa21+ Xa13*+xa5	76	3	3	94.51
HR 23-65-6-38	xa21+ xa13+xa5	76	3	3	94.51
HR 23-65-6-142	Xa 21+ xa13+ xa5	76	2	4	93.90
HR 23-65-6-191	Xa 21+ xa13+ xa5	75	3	4	93.29
HR 23-65-6-237	Xa21+ xa13+ xa5	75	4	3	93.90
HR 23-65-6-242	Xa 21+xa13+ Xa5	75	3	4	93.29
HR 23-65-6-256	Xa 21*+ Xa13*+ xa5	77	3	2	95.73
HR 23-65-6-258	Xa21+xa13+xa5	77	2	3	95.12
HR 23-135-83-7	Xa21+Xa13*+xa	76	3	3	94.51
HR 23-135-83-24	Xa21+xa13+xa5	77	2	3	95.12
HR 23-135-83-95	xa21+Xa13+xa5	77	4	1	96.34
AVERAGE		76.07	3.00	2.93	94.59

*denote heteroalleles/segregating alleles

Supplementary Table S5. X^2 analysis for aroma and palatability in RP and improved lines (BC_2F_4) based on panel test

Sample set	No. of panellist	No. of judgments for aroma and palatability test in rice		
		Correct	Incorrect	X^2 value
RP + HR 23-5-37-83-5	40	18	22	1.85
RP + HR 23-5-37-83-12	40	14	26	2.38
RP + HR 23-5-37-121-3	40	14	26	2.38
RP + HR 23-5-37-121-10	40	18	22	1.85
RP + HR 23-5-37-121-14	40	17	23	0.49
RP + HR 23-5-37-201-9	40	17	23	0.49
RP + HR 23-65-6-142-3	40	15	25	0.56
RP + HR 23-65-6-142-18	40	17	23	0.49
RP + HR 23-65-6-191-13	40	18	22	1.85
RP + HR 23-65-6-237-2	40	15	25	0.56
RP + HR 23-65-6-237-27	40	15	25	0.56
RP + HR 23-65-6-258-10	40	15	25	0.56
RP + HR 23-65-6-258-21	40	17	23	0.49
RP + HR 23-135-83-24-1	40	17	23	0.49
RP + HR 23-135-83-24-22	40	16	24	0.85

Note: Null hypothesis is rejected as Chi-squire value is not significant, RP line is not significantly differing from NILs for palatability.

Supplementry Table S6. Performance of parents and BC2F4 derivatives of HUR 917/ IRBB66 for 15 morpho-agronomical traits disease severity, AUDPC value and genome recovery

Lines/ NIL no.	DPI	DFPE	DFF	DM	PH	NEBT	PL	GPP	SF	GYPP	GY (kg/ha)	TW	KL	KB	KL BR	HRR	AC%	GC	Aroma	PDI with SD (%)			AUD PC
																				14 DAI	21 DAI	28 DAI	
HUR917	83	100	106	135	112.20	9.50	24.82	195.6	87.59	6.80	2,264.4	14.2	5.04	1.70	2.965	68.5	23.80	28	2	30.6	49.92	58.78	873.35
IRBB66	85	102	107	135	108.50	10.80	22.42	192.5	77.61	20.40	6799.32	20.6	6.11	1.82	3.357	72.5	19.60	55	0	3.03	3.38	3.65	74.13
HR 23-5-37-83-5	86	103	108	136	106.05	9.25	20.62	188.5	84.32	19.20	6399.36	16.4	6.12	1.86	3.308	68.6	22.73	35	2	2.15	3.36	2.68	77.25
HR 23-5-37-83-12	88	105	110	130	104.01	8.75	22.42	200.1	82.33	18.60	6199.38	18.6	6.31	1.69	3.734	58.6	20.18	39	2	5.12	7.91	9.31	105.88
HR 23-5-37-121-3	85	100	106	132	102.14	8.65	24.00	210.6	86.33	20.20	6732.66	20.2	6.22	1.74	3.575	62.4	21.62	28	2	3.42	4.08	2.81	59.89
HR 23-5-37-121-10	90	106	110	135	107.05	9.12	25.40	204.5	85.46	21.40	7132.62	14.2	5.32	1.71	3.111	65.8	22.87	36	2	2.89	4.69	2.98	64.16
HR 23-5-37-121-14	87	104	108	125	101.71	10.00	22.82	201.6	88.89	16.20	5399.46	13.6	5.08	1.70	2.988	76.5	24.12	38	2	6.20	7.03	8.86	122.42
HR 23-5-37-201-9	83	98	106	125	106.38	12.40	23.65	198.0	88.13	17.50	5832.75	18.5	5.80	1.68	3.452	67.0	20.09	33	2	4.28	4.86	6.42	98.52
HR 23-65-6-142-3	91	108	112	135	104.41	10.20	24.08	190.6	89.13	18.00	5999.40	20.1	6.04	1.85	3.265	64.2	21.82	34	2	2.81	4.19	1.83	36.07
HR 23-65-6-142-18	88	102	108	130	106.91	9.80	25.00	189.2	90.23	16.80	5599.44	20.0	6.42	1.78	3.607	59.2	20.47	51	2	2.89	3.87	4.65	53.48
HR 23-65-6-191-13	90	105	110	140	103.03	9.08	22.68	192.7	88.13	16.50	5499.45	16.4	5.42	1.72	3.151	53.6	23.66	55	2	6.25	7.42	8.05	154.5
HR 23-65-6-237-2	84	100	105	135	105.13	9.32	23.65	185.5	87.86	20.08	6692.66	18.2	5.68	1.74	3.264	78.4	23.47	31	2	2.32	3.28	3.01	65.12
HR 23-65-6-237-27	82	98	104	125	106.02	10.26	22.42	185.2	90.50	19.60	6532.68	20.0	6.02	1.82	3.308	69.5	21.85	45	2	3.57	5.19	8.32	77.95
HR 23-65-6-258-10	86	103	109	132	104.32	8.06	21.54	190.5	76.20	18.20	6066.06	16.4	5.08	1.79	2.838	73.1	22.78	36	2	6.27	7.13	8.19	100.52
HR 23-65-6-258-21	86	100	106	130	103.62	8.90	20.60	195.6	88.40	17.60	5866.08	15.6	5.40	1.67	3.234	79.2	20.12	38	2	3.03	4.76	5.87	64.47

HR 23-135-83-24-1	90	104	110	135	102.52	10.80	25.08	202.4	82.50	16.00	5332.80	17.2	5.92	1.82	3.253	63.5	24.08	44	2	6.07	7.18	9.12	103.43
HR 23-135-83-24-22	91	106	110	135	109.47	11.24	23.60	208.2	88.86	19.40	6466.02	20.3	6.49	1.78	3.646	60.2	22.67	59	2	4.23	7.25	9.12	97.48
Mean	86.76	102.59	107.94	132.35	105.50	9.77	23.22	195.96	86.03	18.38	6126.05	17.68	5.79	1.76	3.30	67.11	22.11	40.29	1.88	5.60	7.97	9.04	131.10

Note: *NIL-near isogenic lines, DPI-days to panicle initiation, DFPE-days to first panicle emergence, DFF-days to 50% flowering, DM-days to maturity, PH-plant height, NETPP-number of effective tillers per plant, PL-panicle length, NGP-number of grains per panicle, TW-test weight, GYPP-grain yield per plant, KL-kernel length, KB-kernel breadth, KLBR-kernel length/breadth ratio, HRR-head rice recovery, AC-amylose content, GC-gelatinization content and DS-disease severity; DAI-days after inoculation and AUDPC-area under disease progress curve; GR-genome recovery.*

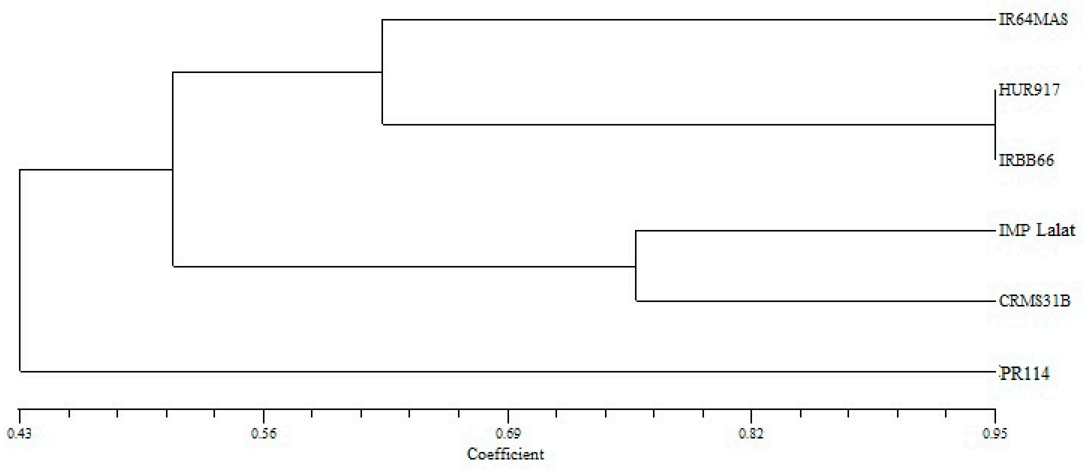

Supplementary Figure S1. Genetic relationship amongst the recurrent parent HUR 917 and donors

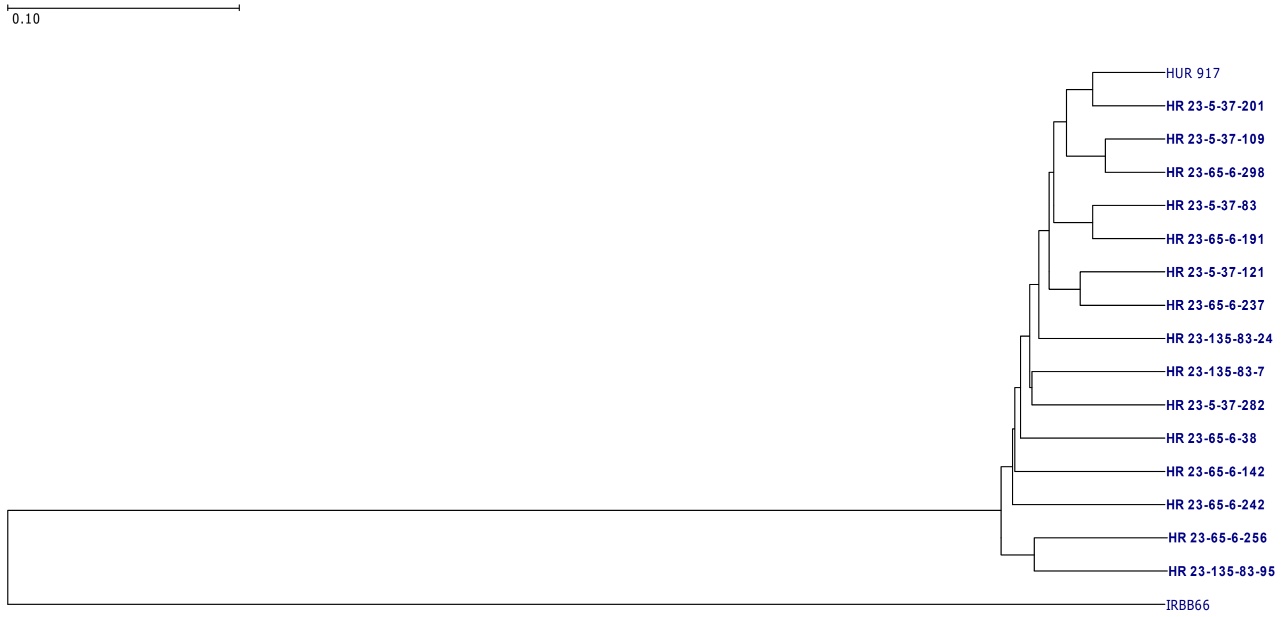

Supplementary Figure S2. Genetic relationship amongst the recurrent parent HUR 917 and NILs

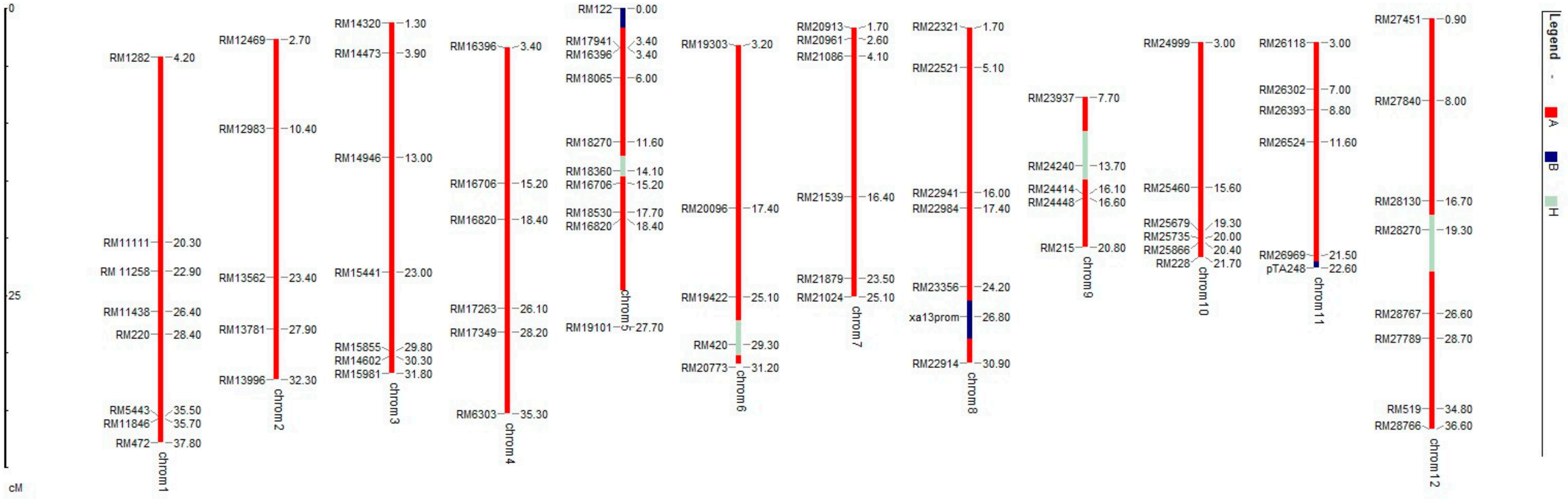

Supplementary Figure S3. Physical position of SSR markers used for gene introgression and genome recovery of the recurrent parent.

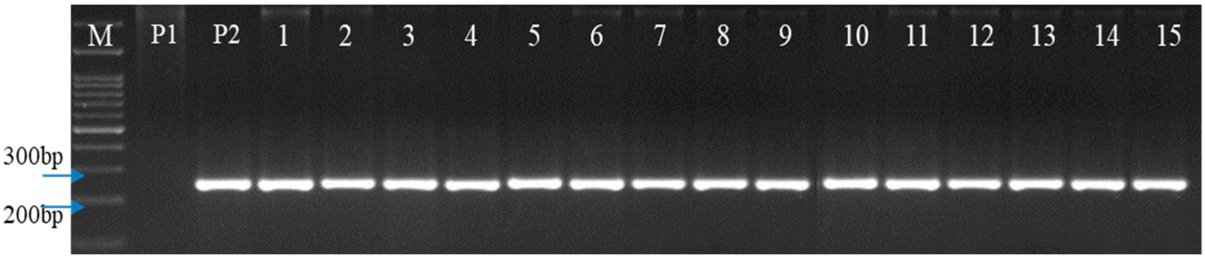

Supplementary Figure S4. PCR amplification of BADH2 gene in BC_2F_4 NILs using FMbadh2-E7 functional marker; M, Marker; P1- IRBB66, P2-RP (HUR 917) and lane 3-18 are NILs.

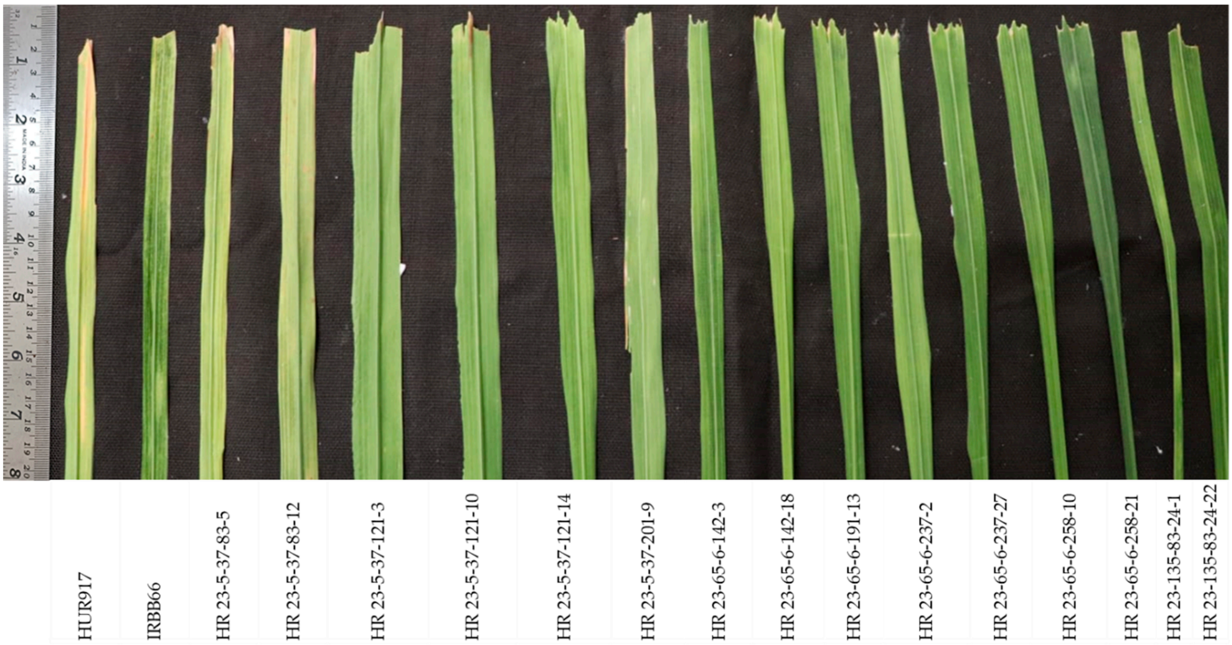

Supplementary Figure S5. Bacterial blight disease severity in parents and NILs after 21 days of inoculation of eight virulent *Xoo* races.

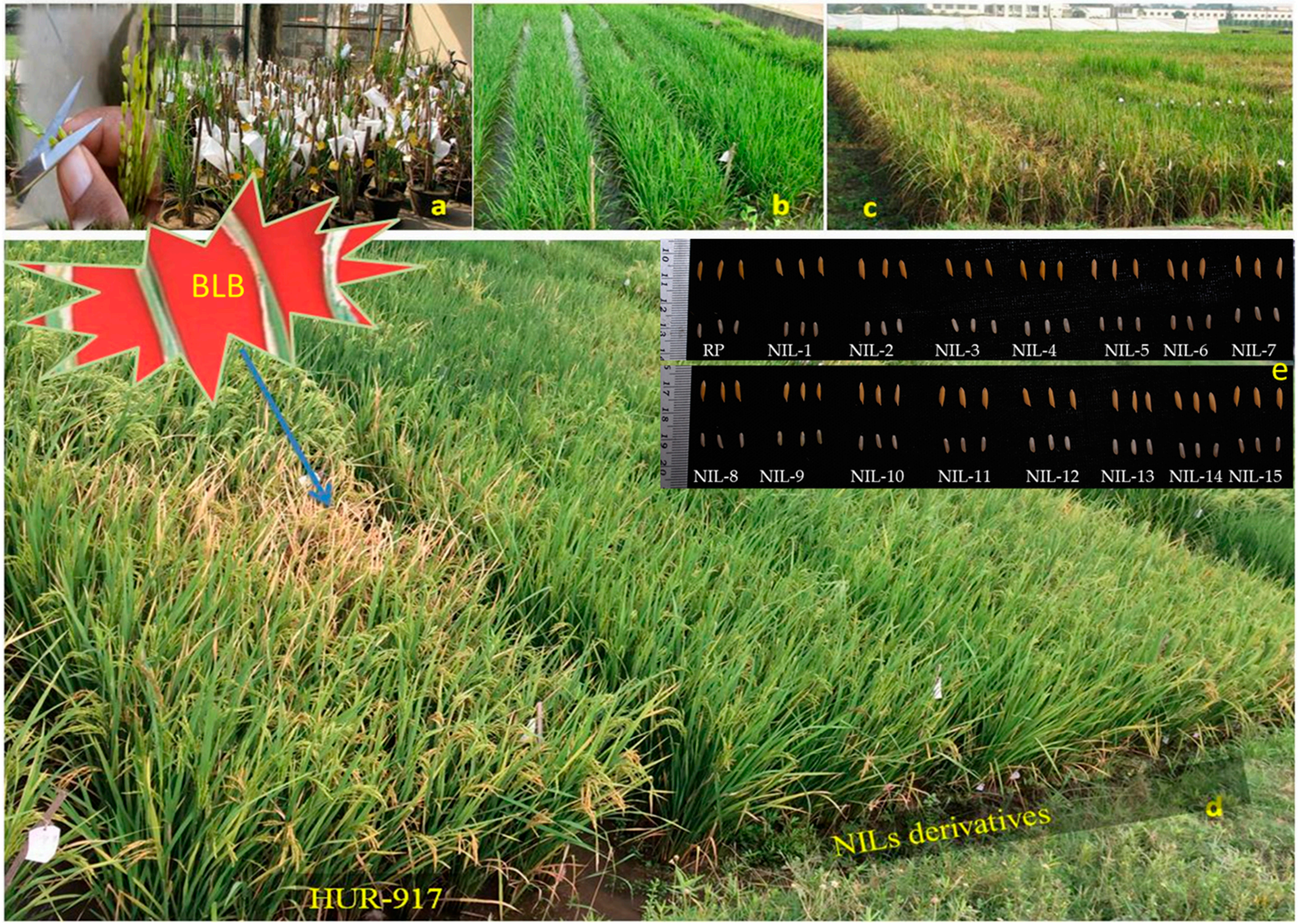

Supplementary Figure S6. Field view parents and NILs of HUR 917/IRBB66. (a) Crosses generation. (b) Preliminary evaluation. (c) Morphological variation in NILs. (d) Field view under natural condition. (e) Grain size of parents and NILs.
